# Using Fatty Acid-Binding Proteins as Potential Biomarkers to Discriminate between Parkinson’s Disease and Dementia with Lewy Bodies: Exploration of a Novel Technique

**DOI:** 10.3390/ijms241713267

**Published:** 2023-08-26

**Authors:** Ichiro Kawahata, Tomoki Sekimori, Hideki Oizumi, Atsushi Takeda, Kohji Fukunaga

**Affiliations:** 1 Department of CNS Drug Innovation, Graduate School of Pharmaceutical Sciences, Tohoku University, Sendai 980-8578, Japankfukunaga@tohoku.ac.jp (K.F.); 2 Department of Neurology, National Hospital Organization Sendai Nishitaga Hospital, Sendai 982-0805, Japantakeda.atsushi.nc@mail.hosp.go.jp (A.T.)

**Keywords:** fatty acid-binding proteins, biomarkers, Parkinson’s disease, dementia with Lewy bodies, α-synucleinopathies, dementia, diagnosis, discrimination technique

## Abstract

An increase in the global aging population is leading to an increase in age-related conditions such as dementia and movement disorders, including Alzheimer’s disease (AD), Parkinson’s disease (PD), and dementia with Lewy bodies (DLB). The accurate prediction of risk factors associated with these disorders is crucial for early diagnosis and prevention. Biomarkers play a significant role in diagnosing and monitoring diseases. In neurodegenerative disorders like α-synucleinopathies, specific biomarkers can indicate the presence and progression of disease. We previously demonstrated the pathogenic impact of fatty acid-binding proteins (FABPs) in α-synucleinopathies. Therefore, this study investigated FABPs as potential biomarkers for Lewy body diseases. Plasma FABP levels were measured in patients with AD, PD, DLB, and mild cognitive impairment (MCI) and healthy controls. Plasma FABP3 was increased in all groups, while the levels of FABP5 and FABP7 tended to decrease in the AD group. Additionally, FABP2 levels were elevated in PD. A correlation analysis showed that higher FABP3 levels were associated with decreased cognitive function. The plasma concentrations of Tau, GFAP, NF-L, and UCHL1 correlated with cognitive decline. A scoring method was applied to discriminate between diseases, demonstrating high accuracy in distinguishing MCI vs. CN, AD vs. DLB, PD vs. DLB, and AD vs. PD. The study suggests that FABPs could serve as potential biomarkers for Lewy body diseases and aid in early disease detection and differentiation.

## 1. Introduction

The global aging population is experiencing rapid growth. It is expected that by 2050, the number of individuals aged 65 years and older will be more than double that of the current population. This demographic shift has increased the prevalence of age-related conditions such as dementia and movement disorders, including Alzheimer’s disease (AD), Parkinson’s disease (PD), and dementia with Lewy bodies (DLB). To effectively address these challenges, it is crucial to accurately predict the risk factors associated with these disorders and provide an early diagnosis, which could prevent age-related diseases, significantly enhance healthcare, improve living standards, and extend the life expectancy of individuals affected by these conditions.

Biomarkers are biological molecules used to diagnose and monitor various diseases and conditions. Biomarkers can help identify and track changes in the brain that are linked to dementia and movement disorders. These biomarkers are typically found in bodily fluids, such as blood or cerebrospinal fluid, and can provide prospective information about the presence and severity of a particular condition. For instance, altered levels of specific proteins in the blood and cerebrospinal fluid are associated with the development of certain diseases.

α-Synucleinopathy is a term used to describe a group of neurodegenerative disorders characterized by the presence of abnormal deposits of a protein called α-synuclein in the brain. These disorders include PD, DLB, and multiple system atrophy. α-Synuclein is distributed in neuronal cells in the brain and forms aggregates called Lewy bodies in PD and DLB. Previous studies have investigated the role of plasma α-synuclein levels in the development of these disorders. However, these findings are inconsistent, with some studies reporting an increase in plasma α-synuclein levels and others reporting a decrease [1]. Therefore, additional conditions are required to reflect the pathology of Lewy body disease. Additionally, DLB and Parkinson’s disease dementia (PDD) are Lewy body-related neurodegenerative disorders that share common clinical and neuropathological characteristics [2], making it crucial to develop techniques capable of distinguishing them during the early stages of disease progression.

In AD, it is essential to predict the disease by analyzing biomarkers. Biomarkers for AD include the protein Tau, β-amyloid, and neurofilament light (NF-L) in the cerebrospinal fluid, as well as Tau and β-amyloid in positron emission tomography (PET) imaging. These biomarkers are used to diagnose AD, track the progression of the disease, and assess treatment efficacy. However, there are still some problems with the use of biomarkers for diagnosing and treating AD, including the need for invasive procedures to collect cerebrospinal fluid and the cost of PET imaging. Regarding PET, there are concerns about radiation exposure given its long-term use in disease progression and the need for re-evaluation. In terms of specificity, the direct observation of abnormalities in specific proteins or neural pathways requires a combination of other techniques.

The analysis of plasma biomarkers offers a simple and cost-effective method of examination. However, further investigation is needed to fully understand the physiological significance of these plasma biomarkers. We previously identified type 3 fatty acid-binding protein (FABP3) within Lewy bodies in an autopsy of the brain of a patient with PD [3]. FABP3 is highly expressed in dopaminergic neurons [4] and directly interacts with α-synuclein [5], a pathogenic protein in PD. FABP3 is critical for facilitating the uptake, propagation, and neurotoxicity of α-synuclein in neuronal cells [4,6,7,8]. Notably, FABP3 colocalizes with α-synuclein aggregates and contributes to mitochondrial dysfunction and the loss of tyrosine hydroxylase, a rate-limiting enzyme in dopamine synthesis [4,9]. Encouragingly, the inhibition of FABP3 using small-molecule ligands and peptides has demonstrated the ability to prevent FABP3-induced neurotoxicity [6,10,11,12]. These findings highlight FABP3 as a potential target for addressing α-synuclein pathology and its associated effects.

Other fatty acid-binding proteins (FABPs) that may be involved in α-synucleinopathies include FABP5 and FABP7. FABP5 can modulate the inflammatory response and the production of reactive oxygen species (ROS) in glial cells, which may affect neuronal survival and α-synuclein aggregation [13,14,15]. Additionally, FABP7 is expressed in oligodendrocytes, which are myelin-producing cells that accumulate α-synuclein and contribute to Lewy body disease pathology [16,17,18]. FABP7 regulates the lipid composition of myelin and influences its stability and function. Therefore, FABPs may be potential biomarkers for α-synucleinopathies as they can reflect lipid status, the inflammatory state, and neuronal or glial damage caused by an α-synuclein pathology. Furthermore, FABPs may also be therapeutic targets for α-synucleinopathies because modulating their expression or activity may affect the uptake, propagation, and toxicity of α-synuclein in the brain. These data suggest that FABP family proteins are responsible for the pathogenesis of Lewy body diseases and are potential biochemical biomarkers for discriminating healthy individuals.

Based on previous studies, the current study aimed to investigate the correlation between modifications in plasma FABP levels and each neurodegenerative disease using noninvasive measurements of human plasma biomarkers. We also aimed to determine whether these markers are helpful in discriminating between specific neurodegenerative diseases.

## 2. Results

### 2.1. FABP3 as a Potential Biomarker for Lewy Body Diseases

**Figure 1:**
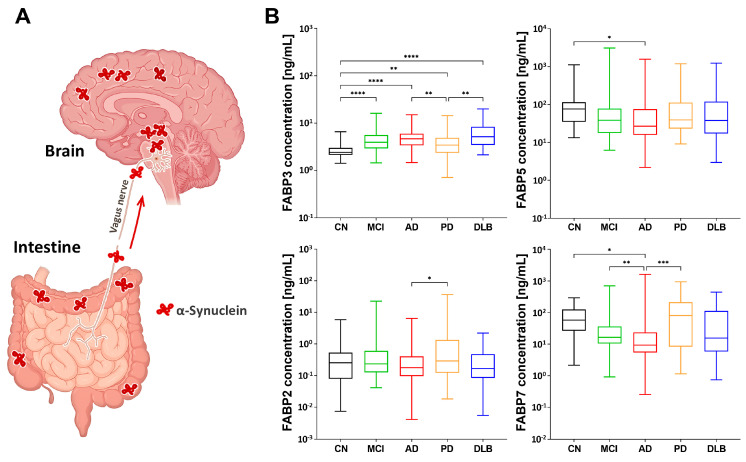
The propagation of pathogenic α−synuclein and the modulation of plasma fatty acid−binding proteins (FABPs). (**A**) Schematic diagram illustrating the gut−to−axis propagation of pathogenic proteins, specifically α−synuclein, in Lewy bodies. (**B**) Alterations in plasma levels of FABP2, FABP3, FABP5, and FABP7 in different groups, including healthy controls (CN in black color), patients with mild cognitive impairment (MCI in green), Alzheimer’s disease (AD in red), Parkinson’s disease (PD in orange), and Dementia with Lewy bodies (DLB in blue). Data were analyzed using the Kruskal–Wallis test, followed by Dunn’s multiple comparison test. * *p* < 0.05, ** *p* < 0.01, *** *p* < 0.001, and **** *p* < 0.0001.

In Lewy body diseases, the pathogenic protein α-synuclein propagates and FABP3 participates in this process. Additionally, FABP5 is associated with α-synuclein-induced mitochondrial injury, whereas FABP7 is involved in oligodendrocyte degeneration. Furthermore, the gut-to-brain migration of α-synuclein plays a critical role in this disease (Figure 1A). With this background, we investigated whether the FABP family proteins have the potential to reflect the state of Lewy body diseases.

Here, we measured the plasma levels of FABPs in patients with AD, PD, DLB, and MCI and compared them with those in the CN group using a single-molecule array Simoa. We found that the plasma levels of FABP3 increased in all groups, including the MCI group (Figure 1B). However, the FABP5 and FABP7 levels tended to decrease in the AD group. FABP2, which is predominantly expressed in the small intestine and is involved in the absorption and intracellular transport of dietary fatty acids, was significantly increased in the PD group compared to AD. Overall, these findings suggest that FABP3 can be a potential biomarker for these diseases. However, it is difficult to distinguish each disease from the others based on these data alone.

### 2.2. Distinctive Disease Profiling through Established Biomarkers

**Figure 2:**
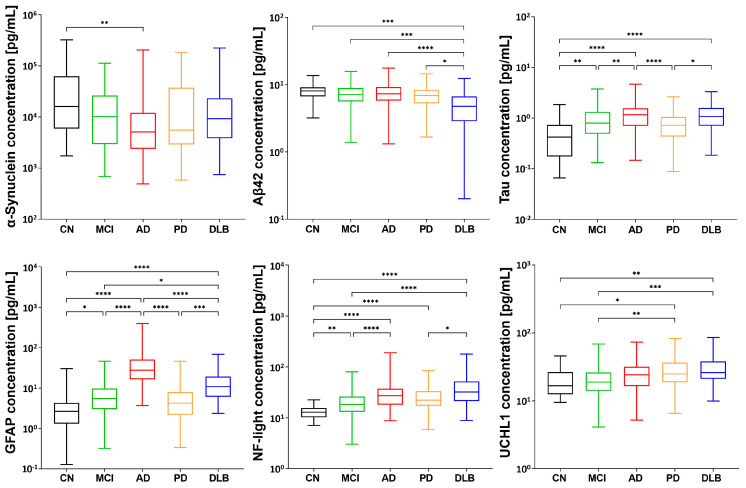
Alterations in the plasma levels of general biomarkers. The figure illustrates the plasma concentrations of α-synuclein, amyloid β 42 (Aβ42), total Tau, GFAP, NF-L, and UCHL1, highlighting the pathological changes associated with these biomarkers. Data were analyzed via the Kruskal–Wallis test, followed by Dunn’s multiple comparison test. * *p* < 0.05, ** *p* < 0.01, *** *p* < 0.001, and **** *p* < 0.0001. In the figure, CN is shown in black, MCI in green, AD in red, PD in orange, and DLB in blue.

We then measured the known biomarkers and conducted a correlation analysis. Using Simoa, we observed a decrease in plasma α-synuclein levels across all groups, with a significant decrease observed in the AD group. Additionally, we observed a decrease in the levels of Aβ42, a protein implicated in AD, in the DLB group (Figure 2). An analysis of the levels of Tau revealed an increase in all groups, whereas the level of phosphorylated Tau exhibited wide variability without significant differences. We found that GFAP, a biomarker associated with brain injury and astrocyte activation, tended to increase in all groups and was particularly elevated in the AD group. Similarly, NF-L levels increased in all groups. Lastly, we investigated Ubiquitin C-terminal hydrolase L1 (UCHL1), a protein involved in protein degradation, and observed its elevation in α-synucleinopathies such as PD and DLB, while no significant elevation was observed in the MCI and AD groups.

### 2.3. Correlation Analysis of FABP3 and Established Biomarkers with Cognitive Function

**Figure 3:**
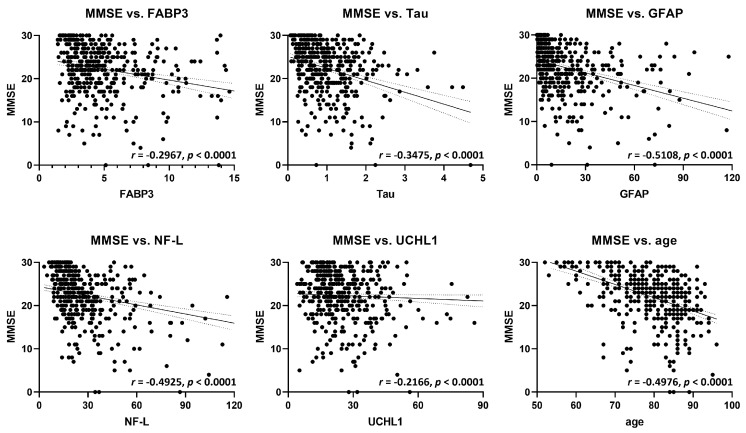
Correlation analysis between various plasma biomarkers and the Mini−Mental State Examination (MMSE). The plasma concentrations of FABP3, total Tau, GFAP, NF−L, and UCHL1 are shown. The correlation diagrams demonstrate the relationship between the MMSE and FABP3, Tau, GFAP, NF−L, and UCHL1, as well as age. Correlation coefficients (R values) ranging from 0.8 to 1.0, from 0.5 to 0.8, and from 0.2 to 0.5 were categorized as strong, moderate, and weak correlations, respectively. In the correlation analyses, the *p*−values for all analyzed groups were *p* < 0.0001.

Subsequently, a correlation analysis was conducted to examine the relationship between FABP3 and other established biomarkers using the MMSE, a measure of cognitive function. The findings revealed a tendency for higher plasma FABP3 levels to be associated with lower MMSE scores (Figure 3). Additionally, a negative correlation was observed between MMSE scores and the plasma concentrations of Tau, GFAP, NF-L, and UCHL1 (Figure 3). Furthermore, a decline in MMSE scores was correlated with aging. Overall, these results indicate the potential usefulness of these biomarkers for predicting the progression of cognitive decline in each disease. The *p*-values for all analyzed groups were *p* < 0.0001, indicating the validity of the analysis. However, further improvements in accuracy are required for the precise identification of each specific disease.

### 2.4. Multimarker Score for Accurate Disease Discrimination and Differential Diagnosis

Next, we attempted to calculate a score utilizing the plasma levels of multiple biomarkers, including FABPs, to distinguish each disease. Initially, we explored a method with a high degree of accuracy for discriminating between healthy subjects and those with diseases. The analysis revealed that the obtained figures could accurately infer the presence of disease in healthy subjects, as well as in those with AD, PD, and DLB, with a remarkable accuracy of *p* < 0.0001 (Supplemental Figure S1A, AUC > 0.9; Supplemental Figure S1B). However, further improvements are necessary to enhance the accuracy of the discrimination of individual diseases.

**Figure 4:**
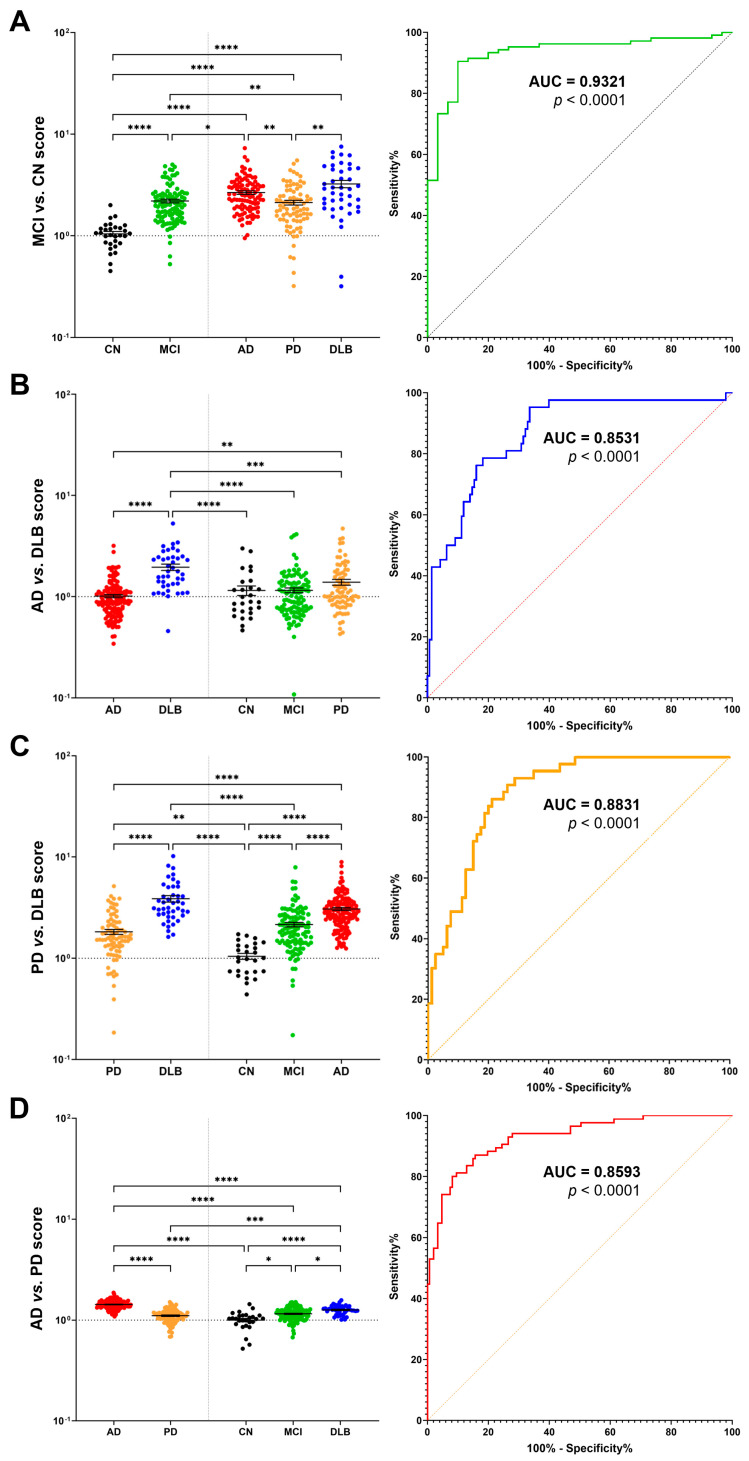
Comparison of score values and accuracy for each quantified disease group. The figure displays the comparison of quantified score values for different disease groups, including (**A**) MCI vs. CN, (**B**) AD vs. DLB, (**C**) PD vs. DLB, and (**D**) AD vs. PD, as well as within each individual group. In the right column, the accuracy of the quantified score values was assessed using receiver operating characteristic (ROC) curves. ROC curves for (**A**) MCI vs. CN, (**B**) AD vs. DLB, (**C**) PD vs. DLB, and (**D**) AD vs. PD are presented, demonstrating the performance and discriminative ability of the quantified scores. Data were analyzed via the Kruskal–Wallis test, followed by Dunn’s multiple comparison test to compare each disease group. * *p* < 0.05, ** *p* < 0.01, *** *p* < 0.001, and **** *p* < 0.0001. In the figure, CN is shown in black, MCI in green, AD in red, PD in orange, and DLB in blue.

Therefore, we calculated a score to differentiate between each disease. The results showed remarkable accuracy when comparing the MCI vs. CN groups (Figure 4A), AD vs. DLB groups (Figure 4B), PD vs. DLB groups (Figure 4C), and AD vs. PD groups (Figure 4D). In detail, the DLB patients demonstrated higher values than the AD and PD patients (Figure 4B,C), whereas the PD patients exhibited lower values than the AD patients (Figure 4D). The Area Under the Curve (AUC) values were 0.9321 for MCI vs. CN (Figure 4A, right column), 0.8531 for AD vs. DLB (Figure 4B, right column), 0.8831 for PD vs. DLB (Figure 4C, right column), and 0.8593 for AD vs. PD (Figure 4D, right column). Although false negatives are inevitable, the current approach enables the detection of Lewy body disease with greater accuracy than in previous reports.

## 3. Discussion

Several studies have attempted to distinguish between PD, PDD, and DLB using biomarkers in the cerebrospinal fluid and serum concentrations of FABPs [2,19,20,21]. These studies focused on the measurement and analysis of the cerebrospinal fluid and serum FABP3 levels, which revealed distinct changes associated with PD, PDD, and DLB. Increased FABP3 levels were consistently observed in both cerebrospinal fluid and serum samples from patients with PD, PDD, and DLB. Notably, FABP3 levels were higher in DLB than in PDD or AD, indicating the potential of FABP3 as a biomarker specific to DLB. Additionally, Mollenhauer et al. attempted to improve sensitivity and specificity by utilizing the ratios of FABP3 and Tau [22]. Therefore, in this study, we aimed to establish a more accurate predictive technique, using plasma biomarkers to differentiate between PD and DLB and to distinguish them from AD and healthy individuals.

This study provides beneficial insights into the potential of combining FABPs as biomarkers for Lewy body diseases. The intricate involvement of FABPs, particularly FABP3, FABP5, and FABP7, in the pathogenesis of Lewy body diseases highlights their significance in reflecting the disease state. Additionally, we examined the plasma levels of known biomarkers associated with these diseases and conducted correlation analyses to explore their relationships with cognitive function and disease progression.

Our investigation revealed that FABP3 plasma levels were elevated across all groups, including patients with MCI, AD, PD, and DLB, compared to the CN group, which is consistent with the serum levels reported in previous studies [2,20,21,23]. This suggests the potential of FABP3 as a general biomarker of Lewy body disease. In contrast, FABP5 and FABP7 levels tended to decrease in the AD group, indicating potential disease-specific mechanisms associated with these proteins. FABP2, which is predominantly expressed in the small intestine and is involved in fatty acid transport, was preferentially elevated in the PD group, emphasizing its relevance in the pathogenesis of PD. These findings collectively support the notion that FABPs may serve as potential biomarkers for Lewy body diseases, although distinguishing specific diseases based solely on these data remains a challenge.

To further understand disease-related modifications, we analyzed the plasma levels of established biomarkers in correlation with the disease state and cognitive function. Our results showed decreases in plasma α-synuclein levels across all groups, with a significant decrease in the AD group. A decrease in the level of Aβ42, a protein implicated in AD, was also observed in the DLB group. Tau protein levels increased in all groups, whereas phosphorylated Tau levels exhibited variability without significant differences. GFAP, which is associated with brain injury and astrocyte activation, tended to increase in all groups, and it was notably elevated in the AD group. Similarly, NF-L levels increased in all groups. Furthermore, UCHL1, a protein involved in protein degradation, was elevated in the PD and DLB groups but not in the MCI and AD groups. These findings provide additional evidence of the complex molecular alterations associated with Lewy body diseases and their impact on cognitive function.

There is conflicting evidence regarding the plasma levels of α-synuclein in previous reports, with some studies reporting a decrease and others reporting an increase [21,24,25,26,27,28,29,30]. One possible explanation for this discrepancy is the phenomenon of the gut-to-brain propagation of α-synuclein wherein α-synuclein from peripheral sources, including plasma, may migrate and accumulate in the brain, resulting in a decrease in the peripheral α-synuclein levels. Accordingly, the availability of monomeric α-synuclein for normal functions is reduced. Interestingly, it has been reported that the plasma levels of pathogenic α-synuclein seeds increase in synucleinopathies [31]. Further in-depth analyses are required to deepen our understanding of the modification of α-synuclein plasma levels.

Correlation analyses revealed significant associations between FABP3 and established biomarkers with MMSE scores, indicating a potential link between FABP3 levels and cognitive decline. Higher plasma levels of FABP3 and elevated levels of Tau, GFAP, NF-L, and UCHL1 were associated with decreased MMSE scores. Additionally, a decline in MMSE scores was correlated with aging, suggesting the progressive nature of cognitive decline in these diseases. These findings suggest that these biomarkers have potential utility in predicting disease progression and cognitive decline in Lewy body diseases; however, further improvements in accuracy are required to precisely identify each specific disease.

The patient group in the present study exhibited a higher age range (approximately 73.6 to 82.8 years) compared to that of the control group (62.3 years). Therefore, we conducted a further analysis to investigate the correlation between plasma FABP3 levels and the age of disease onset, as well as the duration of affliction (Supplementary Figure S2). The results indicated that there was no significant correlation between the age of disease onset (Supplementary Figure S2A) and the duration of affliction (Supplementary Figure S2B). Furthermore, we employed the Hoehn–Yahr stage to analyze the correlation between the decline in motor function and FABP3 levels. Notably, a correlation was observed between an elevation in the Hoehn–Yahr stage, indicative of the progression of PD, and an increase in the plasma FABP3 level (Supplementary Figure S2C). Based on these findings, the current study suggests that age itself does not play a significant role in the rate of clinical symptom progression or the elevation of FABP3 levels. Looking ahead, we plan to proceed with plasma collection across multiple institutions to align the mean age of the healthy control group with that of the disease group, facilitating further analysis.

To improve the discriminatory power of the biomarkers, we calculated a score using the plasma levels of multiple biomarkers, including FABPs. Our method accurately distinguished healthy subjects from each disease group, including MCI, AD, PD, and DLB. This approach demonstrated remarkable accuracy in inferring the presence of the disease, with an area under the curve (AUC) > 0.9. However, further improvements are necessary to enhance the accuracy of the discrimination of individual diseases.

Finally, we calculated a score that successfully discriminated between the diseases. The results exhibited high accuracy when comparing AD vs. DLB, PD vs. DLB, and AD vs. PD, with significant AUC values. The DLB patients demonstrated higher values than the AD and PD patients, whereas the PD patients exhibited lower values than the AD patients. Although false negatives were unavoidable, our approach enabled the detection of Lewy body diseases with greater accuracy than in previous reports.

In conclusion, our study highlights the potential of FABPs as biomarkers for Lewy body diseases, emphasizing their involvement in disease processes and correlation with cognitive decline. Furthermore, the score calculated using multiple biomarkers shows promise for accurately distinguishing between different diseases within the Lewy body spectrum. These findings contribute to the deepening comprehension aimed at improving diagnostic accuracy and facilitating personalized treatment strategies for individuals affected by Lewy body diseases. Continued research is necessary to refine and validate these biomarkers and scores, potentially leading to enhanced clinical tools for the identification and management of Lewy body diseases.

## 4. Materials and Methods

### 4.1. Study Population

The subjects were consecutively recruited from among outpatients assessed at the National Sendai Nishitaga Hospital in Miyagi, Japan. The classification of each group as MCI, AD, PD, or DLB was based on clinical diagnosis using the Mini-Mental State Examination (MMSE) [32], Clinical Dementia Rating stage [33], and Movement Disorder Society-Sponsored Revision of the Unified Parkinson’s Disease Rating Scale (MDS-UPDRS) Part III [34] to evaluate cognitive and motor impairment. A CN group with normal cognitive and motor functions was selected from the nonconsanguineous caregivers of patients or through local advertisements. The CN subjects underwent screening to ensure no subjective or objective impairment in cognition or motor function and to confirm their full independence in daily activities.

The details of the plasma samples collected from patients and healthy subjects at the National Hospital Organization Sendai Nishitaga Hospital are as follows: patients with clinically confirmed PD (89 patients, mean age 73.6 ± 8.4 years), DLB (47 patients, mean age 82.5 ± 5.8 years), AD (147 patients, mean age 82.8 ± 6.2 years), and mild cognitive impairment (MCI; 111 patients, mean age 77.7 ± 8.3 years) and healthy control (CN) subjects with no cognitive or motor dysfunctions (30 cases, mean age 62.3 ± 5.7 years).

### 4.2. Ethical Approval

All the participants or their legal representatives provided written informed consent. The human experiments conducted in this study adhered to the principles outlined in the Declaration of Helsinki. The research protocols were approved by the Ethics Committees of Tohoku University (approval number PH19-5) and the National Hospital Organization Sendai Nishitaga Hospital (approval numbers 29-3 and 29-10).

### 4.3. Assays

Plasma samples stored at −80 °C were thawed and centrifuged at 10,000× *g* for 5 min. All specimen analyses were conducted using a Simoa HD-X system (Quanterix, Billerica, MA, USA). Prior to the assay, the samples were diluted with the sample diluent provided in each assay kit or the sample diluent in the Homebrew Assay Development Kit (Quanterix). The diluted samples were then applied to plates and assayed individually. The subsequent measurements were performed according to the manufacturer’s instructions provided in the kit (Quanterix).

To measure FABP2, FABP3, FABP5, and FABP7 levels, a custom assay system was developed using the Homebrew Assay. The assay was conducted according to the protocol provided with the Simoa Homebrew Assay Development Kit (#101354, Quanterix). The reagents necessary for the assay were prepared using Pierce EDC (#A35391; Thermo Scientific, Waltham, MA, USA) and EZ-Link NHS-PEG4-Biotin (#A39259; Thermo Scientific). Capture antibodies were coated onto magnetic beads at a concentration of 0.3 mg/mL, whereas detector antibodies were biotinylated at a 1:40 ratio. These customized reagents enabled the specific detection of FABPs in the samples.

### 4.4. Antibodies

Specific antibodies were used to detect and measure FABP levels. Monoclonal antibodies against FABP3 (capture: 8E3; detector: 8B9) were obtained from Dainippon Sumitomo Pharma Co., Ltd. [35]. Mouse monoclonal antibodies against other FABPs were obtained from BioGate (Gifu, Japan). Calibration curves were established for each combination of the capture and detector antibodies. The study utilized the following antibody combinations: FABP2 (capture: 25E6; detector: 7H8), FABP5 (capture: 32E2; detector: 2F11), and FABP7 (capture: 34F5; detector: 25F6). The calibration curves were generated using serially diluted recombinant protein samples. The samples were measured once in the initial trials and in triplicate for the subsequent measurements.

Specific kits were used to quantify the other biomarkers in our study. The Simoa Aβ42 Advantage Kit (#101664; Quanterix) was used to measure amyloid β 42 (Aβ42) levels, whereas the Simoa pTau181 Advantage Kit (#103377; Quanterix) was used to quantify phosphorylated (p)Tau-181. To assess α-synuclein levels, we utilized the Simoa Alpha-Synuclein Discovery Kit (#102233: Quanterix). Furthermore, the Simoa Neurology 4-Plex A Kit (#102153; Quanterix) was used to measure Tau, NF-L, GFAP, and UCHL1 levels. All measurements were performed with strict adherence to the instructions provided by Quanterix (the manufacturer of each kit).

### 4.5. Scoring Method

During the scoring process, the mean and standard deviation were converted into arbitrary values within the range of 0–100 to ensure consistency [36]. A constant was added to prevent negative values and to provide a lower limit for the range [37]. To facilitate comparability between markers, the data were standardized using the mean and unbiased standard deviation of the CN group as a reference. The standardized values for the CN group were set to a mean value (m_CN_) of 5 and a standard deviation (S_CN_) of 1 [38]. A data analysis was performed using R 3.6.3, a statistical software program. The R programming language was used to calculate the optimal combination of biomarkers that maximized the differentiation between the CN subjects and each disease group. The scoring formulas are provided below; the mathematical formulas for MCI vs. CN (1), AD vs. DLB (2), PD vs. DLB (3), and AD vs. PD (4) were employed for scoring.

(FABP2 × FABP3 × Tau)/(FABP5 × αSyn × Aβ42),(1)

(FABP3 × NF-L × FABP5)/(FABP2 × GFAP × Aβ42),(2)

(FABP3 × GFAP × NF-L)/(FABP2 × UCHL1 × Aβ42),(3)

(FABP3 × GFAP × Tau)/(FABP2 × NF-L × 5),(4)

### 4.6. Statistical Analysis

Nonparametric tests were used for disease-specific comparisons. Specifically, the Mann–Whitney test was used to compare two groups, while the Kruskal–Wallis test, followed by Dunn’s multiple comparison test, was used to compare multiple groups. A two-tailed Spearman’s rank correlation coefficient test was used for the correlation analysis. A *p*-value < 0.05 was considered statistically significant. Data analysis and graph generation were performed using GraphPad Prism 9.4.1 (GraphPad Software, La Jolla, CA, USA) and Python 3.8.5.

## Data Availability

Not applicable.

## Supplementary Materials

The following supporting information can be downloaded at: https://www.mdpi.com/article/10.3390/ijms241713267/s1, Supplemental Figure S1, Comparison of score values and accuracy of quantified data for AD, PD, and DLB, compared with the CN group, and Supplemental Figure S2, Correlation analysis between FABP3 and age of onset, duration, and Hoehn-Yahr stage in PD.
