# Supplementary material for: Using Fatty Acid-Binding Proteins as Potential Biomarkers to Discriminate between Parkinson’s Disease and Dementia with Lewy Bodies: Exploration of a Novel Technique
Source: Int J Mol Sci. 2023 Aug 26;24(17):13267. doi: 10.3390/ijms241713267 (PMC10487513; doi:10.3390/ijms241713267)
Supplement: Supplementary file 1 [file ijms-24-13267-s001.zip › ijms-2552047-supplementary.pdf]

Supplementary Figures

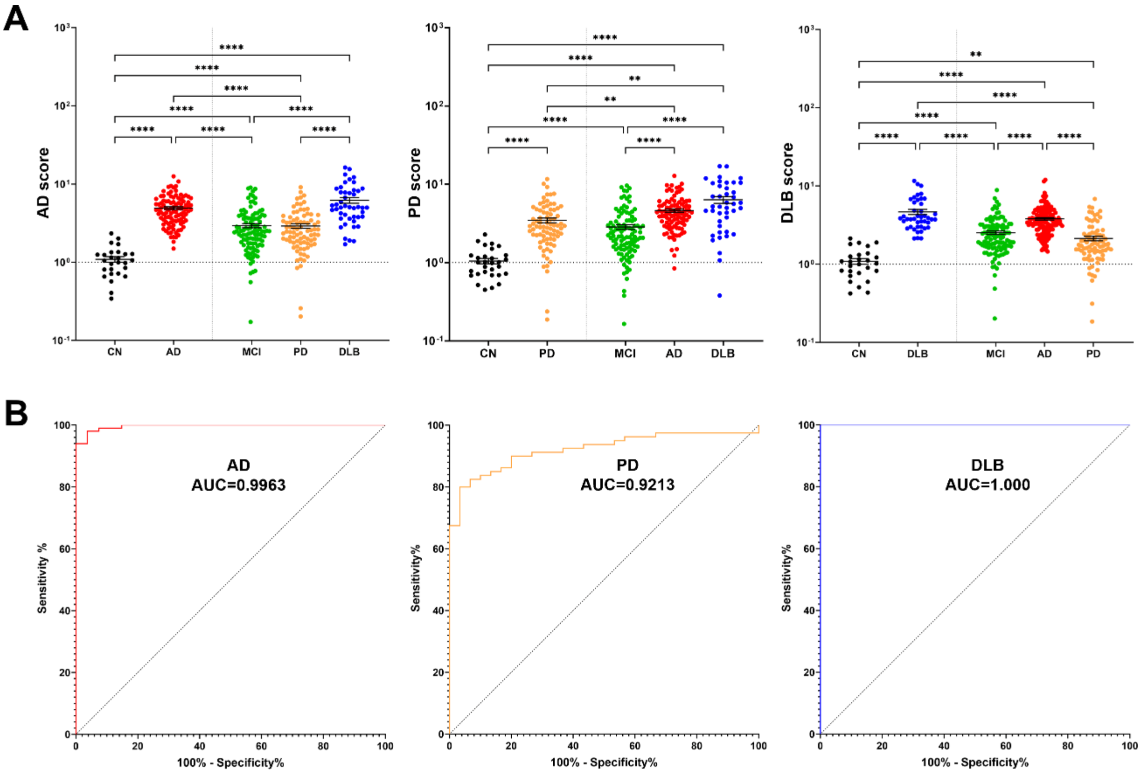

Supplementary Figure S1. Comparison of score values and accuracy of quantified data for each disease compared with the CN group. (**A**) The figure shows the comparison of quantified score values for CN versus different disease groups: AD, PD, and DLB. Data were analyzed using the Kruskal–Wallis test followed by Dunn's multiple comparison test. * $p < 0.05$, ** $p < 0.01$, *** $p < 0.001$, and **** $p < 0.0001$. (**B**) The accuracy of the quantified score values was assessed using ROC curves. ROC curves for CN versus each disorder are shown, demonstrating the performance and discriminative ability of the quantified scores.

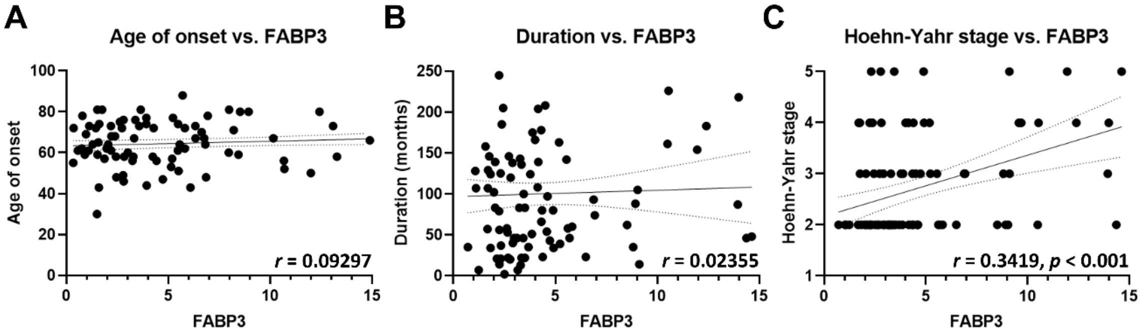

Supplementary Figure S2. Correlation analysis between FABP3 and age of onset (**A**), duration (**B**), and Hoehn-Yahr stage in PD (**C**). In the correlation analyses, the p-value for the FABP3 *vs*. Hoehn-Yahr stage group was p < 0.001. Other groups showed no significance due to the variability in duration and onset age.

Supplementary Data

The mathematical formulas for AD *vs*. CN (1), PD *vs*. CN (2), and DLB *vs*. CN (3) are described below.

$$\frac{FABP3 \times GFAP \times NFlight \times Tau}{\alpha Syn \times A\beta 42 \times 5^2} \quad (1)$$

$$\frac{FABP2 \times FABP3 \times NFlight \times Tau \times UCHL1}{FABP5 \times \alpha Syn \times A\beta 42 \times 5^2} \quad (2)$$

$$\frac{FABP3 \times GFAP \times NFlight \times Tau}{FABP2 \times UCHL1 \times A\beta 42 \times 5} \quad (3)$$
